# Metformin and Atrial Fibrillation: A Systematic Review of Their Association

**DOI:** 10.7759/cureus.64498

**Published:** 2024-07-13

**Authors:** Mohammad Burhanuddin, Vaishnavi Jamched, Muhammad Haris, Husnain Ali, Muhammad Muaz Mushtaq, Maham Mushtaq, Maryyam Liaqat, Muhammad Junaid Aslam, Syed Faqeer Hussain Bokhari

**Affiliations:** 1 Medicine, Bhaskar Medical College, Hyderabad, IND; 2 Medicine and Surgery, Bhaskar Medical College, Hyderabad, IND; 3 Internal Medicine, King Edward Medical University, Lahore, PAK; 4 Medicine and Surgery, King Edward Medical University, Lahore, PAK; 5 Medicine, Mayo Hospital Lahore, Lahore, PAK; 6 Surgery, King Edward Medical University, Lahore, PAK

**Keywords:** cardioprotective effects, diabetes complications, arrhythmia, antihyperglycemic agents, cardiovascular risk, systematic review, type 2 diabetes mellitus, atrial fibrillation, metformin

## Abstract

Atrial fibrillation (AF) is a common cardiac arrhythmia with a significant impact on patient outcomes and healthcare systems. Given the rising incidence of AF with age and its association with conditions, such as diabetes, there is growing interest in exploring pharmacological interventions that might mitigate AF risk. Metformin, a widely prescribed antihyperglycemic agent for type 2 diabetes mellitus (T2DM), has demonstrated various cardiovascular benefits, including anti-inflammatory and antioxidative properties, leading to speculations about its potential role in AF prevention. This systematic review synthesizes findings from five studies examining the association between metformin use and AF risk in patients with T2DM. The review included a dynamic cohort study, three retrospective cohort studies, and a case report, all sourced from databases, such as PubMed, Embase, and the Cochrane Library. The results are mixed; while some studies suggest that metformin use is linked to a reduced incidence of AF, others report no significant association, particularly in postoperative settings. The largest cohort study highlighted a dose-response relationship, suggesting prolonged metformin use correlates with lower AF risk. Conversely, a case report raised concerns about metformin-induced lactic acidosis potentially triggering AF episodes. The review underscores the heterogeneity in study designs and outcomes, pointing to the need for more robust research to establish causality and clarify underlying mechanisms. Future studies should prioritize prospective designs and explore the pleiotropic effects of metformin on atrial remodeling and electrophysiology to better understand its potential role in AF prevention.

## Introduction and background

Atrial fibrillation (AF) is a common cardiac arrhythmia characterized by irregular and rapid contractions of the atria, leading to an impaired ability to effectively pump blood into the ventricles [1,2]. It is the most prevalent sustained cardiac arrhythmia, affecting an estimated 33.5 million individuals worldwide [3-5]. The prevalence of AF increases with age, affecting approximately 9% of individuals aged 65 years or older [6-8]. The pathophysiology of AF involves a complex interplay of various factors, including electrical remodeling, structural changes in the atria, and autonomic imbalance. Risk factors for the development of AF include age, hypertension, coronary artery disease, heart failure, valvular heart disease, diabetes mellitus, obesity, and excessive alcohol consumption [9]. AF is associated with an increased risk of stroke, heart failure, and overall mortality, significantly impacting patients' quality of life and imposing a substantial burden on healthcare systems [10,11].

Metformin is a biguanide antihyperglycemic agent widely used in the management of type 2 diabetes mellitus (T2DM). It acts primarily by reducing hepatic glucose production, increasing peripheral glucose uptake, and improving insulin sensitivity [12]. Metformin is considered the first-line pharmacological treatment for T2DM due to its efficacy, favorable safety profile, and potential cardiovascular benefits [12]. In addition to its glucose-lowering effects, metformin has been associated with various cardiovascular effects, including improvements in endothelial function, reductions in oxidative stress, and anti-inflammatory properties [13]. These pleiotropic effects have sparked interest in investigating the potential role of metformin in the prevention or management of cardiovascular diseases, such as AF. Given the high prevalence of AF, its substantial impact on patient outcomes, and the widespread use of metformin in the management of T2DM, investigating the potential association between metformin use and the risk of developing AF is of significant clinical importance. Several observational studies have explored this relationship, but the findings have been inconsistent, and the underlying mechanisms remain unclear.

This systematic review aims to comprehensively evaluate the existing literature on the association between metformin use and the risk of AF. The primary objective is to synthesize the available evidence from the literature to determine the potential effect of metformin on the incidence or prevalence of AF. In addition, this review will explore potential mechanisms underlying this association, if any, and identify gaps in the current knowledge to guide future research directions.

## Review

Materials and methods

Search Strategy

A comprehensive literature search was conducted in several electronic databases, which included PubMed, Embase, Cochrane Library, and Web of Science. The search strategy combined relevant keywords and Medical Subject Headings (MeSH) terms related to "metformin," "atrial fibrillation," "arrhythmia," and "diabetes mellitus." The searches were limited to studies published from the inception of the database to April 2024. In addition, manual searches of reference lists from relevant articles and reviews were performed to identify any potentially missed studies.

Eligibility Criteria

In this systematic review, we included trials, observational studies (cohort studies, case-control studies, and cross-sectional studies) and case reports that investigated the association between metformin use and the risk of AF. Studies involving adult participants (≥18 years) of any gender, ethnicity, or comorbidity status were considered. The primary outcome of interest was the incidence or prevalence of AF in relation to metformin use. The review was limited to studies published in the English language, acknowledging English as the dominant language of scientific discourse, which facilitates a consistent and accessible body of evidence for analysis. We excluded studies that did not directly address the association between metformin and AF or those that lacked specific, quantifiable outcomes related to AF. This included studies that focused broadly on diabetes or cardiovascular disease without isolating the effects of metformin on AF. Non-English language publications were also excluded to ensure consistency and reliability in the review process, as were unpublished works and gray literature, such as conference abstracts, to uphold a high standard of evidence quality. Studies relying primarily on animal models were excluded to maintain a focus on human-centered research, which is directly applicable to clinical practice. This ensured that the included studies provided clear, relevant, and actionable insights into the potential relationship between metformin and AF.

Study Selection

Two independent reviewers screened the titles and abstracts of the identified studies for eligibility according to the predefined criteria. Any disagreements were resolved through discussion or consultation with a third reviewer, if necessary. Full-text articles of potentially eligible studies were retrieved and assessed for final inclusion in the review. The study selection process was documented using the Preferred Reporting Items for Systematic Reviews and Meta-Analyses (PRISMA) flow diagram.

Data Extraction

Two authors independently extracted the data using a standardized data extraction form to collect relevant information from the included studies. The extracted data included study characteristics (author, year, study design, sample size, and follow-up duration), participant characteristics (age, gender, and comorbidities), exposure details (metformin dosage and duration of use), outcome measures (incidence or prevalence of AF), and main findings of the study.

Data Synthesis

The extracted data were synthesized using a narrative approach due to the substantial heterogeneity of the included studies. This narrative synthesis will provide a descriptive summary of the findings, highlighting the strengths and limitations of the available evidence.

Results

Study Selection Process

**Figure 1:**
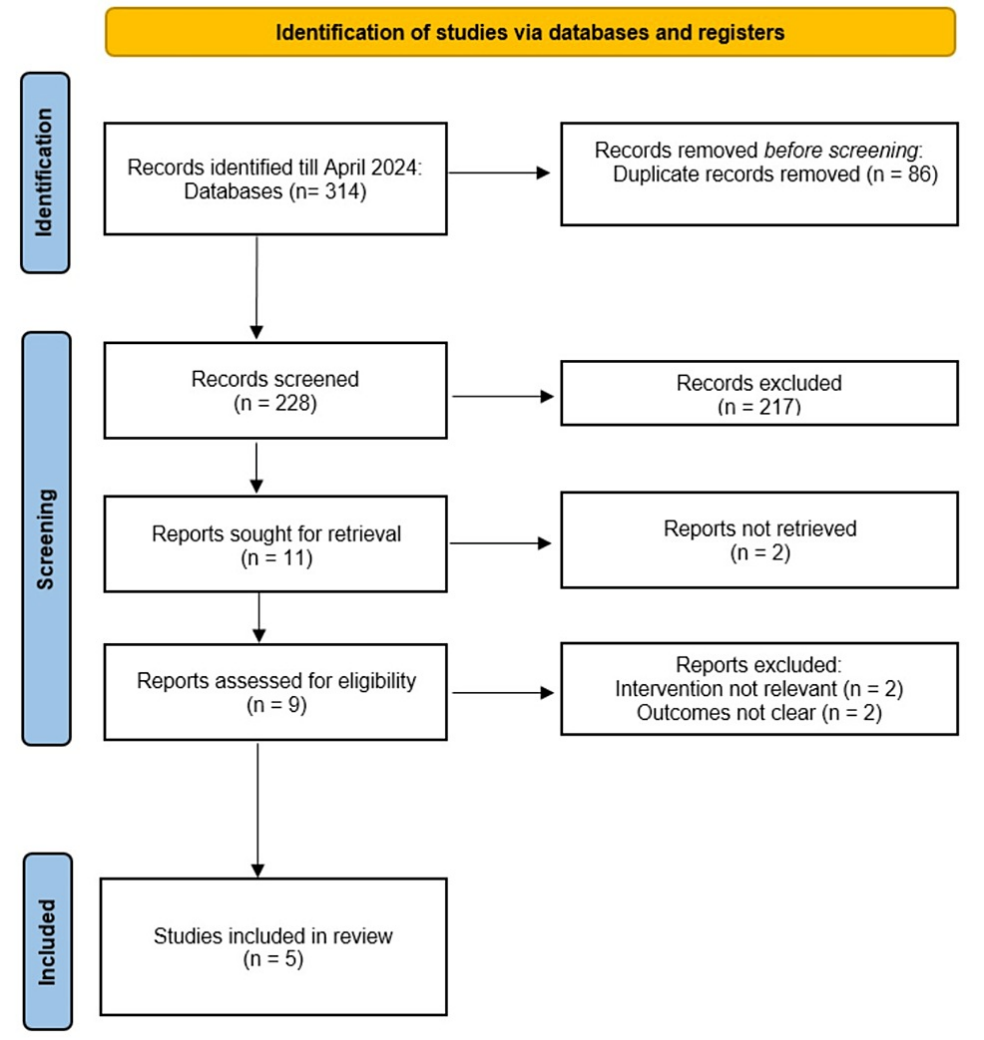
Preferred Reporting Items for Systematic Reviews and Meta-Analyses (PRISMA) diagram illustrating the study selection process.

The database searches initially identified 314 articles. After removing 86 duplicates, the titles and abstracts of the remaining 228 publications were evaluated. Subsequently, 11 studies underwent full-text eligibility assessment. Among these, five articles met the inclusion criteria and were selected for the systematic review. No additional eligible studies were identified through reference list screening of the selected articles. The study selection process is summarized in the PRISMA flow diagram (Figure 1).

Study Characteristics

The systematic review included five studies investigating the association between metformin use and AF in patients with T2DM. The studies were conducted in the USA and Taiwan. The study designs comprised retrospective cohort studies (two from the USA and one from Taiwan), a dynamic cohort/in vitro cell culture study (Taiwan), and a case report (USA). The sample sizes ranged from a single case report to a large cohort of 645,710 participants in the dynamic cohort study by Chang et al. [14,15]. The study population primarily consisted of adults with newly diagnosed T2DM. The majority of the studies included participants of both sexes, with a slight predominance of males in some studies (Table 1).

**Table 1:** Study characteristics of the included studies. T2DM: type 2 diabetes mellitus, AF: atrial fibrillation, CABG: coronary artery bypass graft, HAF: hospitalization for atrial fibrillation, AMRAF: association between first-line monotherapy with metformin and the risk of atrial fibrillation

Title	Metformin Associated Atrial Fibrillation – A Case Report	Association of Metformin With Lower Atrial Fibrillation Risk Among Patients With Type 2 Diabetes Mellitus: A Population-Based Dynamic Cohort and In Vitro Studies	Metformin Therapy and Postoperative Atrial Fibrillation in Diabetic Patients After Cardiac Surgery	Metformin Use Is Associated With a Lower Incidence of Hospitalization for Atrial Fibrillation in Patients With Type 2 Diabetes Mellitus	Association Between First-Line Monotherapy With Metformin and the Risk of Atrial Fibrillation (AMRAF) in Patients With Type 2 Diabetes
Authors	Boolani et al. [14]	Chang et al. [15]	Basnet et al. [16]	Tseng et al. [17]	Iqbal et al. [18]
Publication year	2011	2014	2017	2021	2022
Journal	Journal of Atrial Fibrillation	Cardiovascular Diabetology	Journal of Intensive Care	Frontiers in Medicine	Journal of Diabetes and Its Complications
Country	USA	Taiwan	USA	Taiwan	USA
Study design	Case Report	Dynamic cohort study and in vitro cell culture study	Retrospective Cohort	Retrospective cohort	Retrospective cohort
Study duration	N/A	1999-2010 (13 years of follow-up)	January 1, 2009 to November 30, 2014	1999-2011	2007-2017
Sample size	1	645,710	1,283 patients (635 in the metformin group, 648 in the non-metformin group after matching 114 in each group)	173,398 metformin ever users and 21,666 never users in the unmatched cohort; 21,662 pairs of matched ever and never users	5,664 patients (4,584 on metformin, 1,080 on other monotherapy)
Population	68-year-old male patient	Adults (age ≥18 years) with newly diagnosed T2DM in Taiwan	Diabetic patients aged ≥18 undergoing coronary artery bypass graft (CABG) and/or cardiac valve surgery	Patients with newly diagnosed T2DM	Adults with newly diagnosed T2DM
Age	68	Mean age 58.6 ± 17.1 years	Mean age 65.1 years in the metformin group, 66.6 years in the non-metformin group	Mean age around 64-69 years	Median 56.7 years (metformin group), 66.1 years (other group)
Sex	Male	49.8% male, 50.2% female	70.6% male in metformin group, 65.3% male in non-metformin group	Around 53-54% men	59.9% female (metformin group), 52.3% female (other group)

The baseline characteristics of the study populations varied across the studies, but commonly included comorbidities such as hypertension, dyslipidemia, cardiovascular diseases, and kidney diseases. Some studies provided detailed information on baseline characteristics and matched or adjusted for these factors between the metformin and non-metformin groups. The interventions investigated were metformin therapy, either as monotherapy or in combination with other antidiabetic medications. The dosage and frequency of metformin administration were generally not reported in most studies [14,15,17,18]. The primary outcomes of interest were the incidence, recurrence, and progression of AF in patients with T2DM receiving metformin therapy. Secondary outcomes assessed in some studies included acute renal failure, readmission rates, and all-cause mortality related to AF. The main findings from the studies were mixed. While some studies reported a decreased risk of new-onset AF associated with metformin use, others found no significant association between metformin therapy and postoperative AF incidence [15-18]. The case report by Boolani et al. (2011) highlighted a potential link between metformin-induced lactic acidosis and recurrent AF episodes [14].

Several studies noted a dose-response relationship, with longer durations of metformin use or higher cumulative doses being associated with a more substantial reduction in AF risk (Tseng et al., 2021). However, some studies did not find a statistically significant association between metformin monotherapy and the risk of developing AF compared to other non-insulin monotherapies [18]. A summary of the main findings of included studies is given below (Table 2).

**Table 2:** Summary of the main findings of included studies. AF: atrial fibrillation, T2DM: type 2 diabetes mellitus, CI: confidence interval

Authors	Boolani et al. [14]	Chang et al. [15]	Basnet et al. [16]	Tseng et al. [17]	Iqbal et al. [18]
Intervention Type	Metformin therapy	Metformin therapy	Metformin therapy	Metformin therapy	Metformin monotherapy vs. other non-insulin monotherapy
Dosage	Not mentioned	Not mentioned	≥500 mg	Not mentioned	Not mentioned
Frequency	Not mentioned	Not mentioned	Not mentioned	Not mentioned	Not ,entioned
Comorbidities	T2DM, hypertension, anxiety disorder, coronary heart disease, hyperlipidemia	Hypertension, congestive heart failure, chronic kidney disease, asthma, hyperthyroidism, myocardial infarction, ischemic stroke, sleep apnea syndrome, peripheral arterial disease	Diabetes	Hypertension, dyslipidemia, nephropathy, etc.	Hypertension, hyperlipidemia, coronary artery disease, heart failure, nephropathy, neuropathy, peripheral artery disease, retinopathy, stroke
Main outcomes	The patient developed recurrent AF with repeated administration of metformin, which resolved upon discontinuation of the drug.	After 13 years of follow-up, metformin use was associated with a decreased risk of new-onset AF in patients with T2DM (hazard ratio 0.81, 95% CI 0.76-0.86, p < 0.0001)	Incidence of postoperative AF: 23.5% in the metformin group vs. 26.5% in the non-metformin group before matching (p = 0.2088), 26.3% in the metformin group vs. 30.7% in the non-metformin group after matching (p = 0.4658)	Incidence of hospitalization for atrial fibrillation (HAF). The hazard ratio for ever vs. never users was 0.405 (95% confidence interval: 0.319–0.515) in the unmatched cohort and 0.617 (0.441–0.864) in the matched cohort. Hazard ratios for the tertiles of cumulative duration of metformin therapy vs. never users showed a dose-response effect.	10-year cumulative incidence of AF 5.2% with metformin vs. 8.1% with other agents (not statistically significant)
Secondary outcomes	None specified	None specified	Acute renal failure (0.5% metformin vs. 0.8% non-metformin before matching), 30-day readmission with arrhythmia (0.6% metformin vs. 0.5% non-metformin before matching)	None Specified	All-cause mortality higher in the other group (5.5% vs. 1.7% with metformin)
Main findings	Metformin-induced AF in a patient with each repeated challenge, potentially due to lactic acidosis caused by metformin despite normal hepatic and renal function. The authors hypothesize that the acidic milieu resulting from lactic acidosis could make the heart, particularly the atria, more prone to arrhythmias.	Metformin use was associated with a decreased risk of AF in patients with T2DM who were not using other anti-diabetic medication, probably via attenuation of atrial cell tachycardia-induced myolysis and oxidative stress.	Prior use of metformin therapy in diabetic patients undergoing cardiac surgery was not associated with a decreased rate of postoperative AF.	Metformin use was associated with a lower risk of hospitalization for AF in patients with T2DM, showing a dose-response relationship. The beneficial effect was especially remarkable when metformin had been used for over 2 years.	Initiating metformin as first-line monotherapy for T2DM, compared to other non-insulin monotherapies, was not associated with decreased risk of developing AF in this retrospective study.

Discussion

The findings of this systematic review provide insights into the potential association between metformin use and the risk of AF in patients with T2DM. The largest study, a dynamic cohort study by Chang et al. (2014), reported a decreased risk of new-onset AF associated with metformin use in patients with T2DM [15]. This finding is supported by the retrospective cohort study by Tseng et al. (2021), which demonstrated a lower incidence of hospitalization for AF among metformin users compared to non-users [17]. Notably, Tseng et al. observed a dose-response relationship, with longer durations of metformin use being associated with a more substantial reduction in AF risk. These findings suggest a potential protective effect of metformin against the development of AF in patients with T2DM. Proposed mechanisms underlying this association include the pleiotropic effects of metformin, such as improvements in endothelial function, reductions in oxidative stress, and anti-inflammatory properties [19-22]. Chang et al. (2014) further explored this mechanism through in vitro studies, suggesting that metformin may attenuate atrial cell tachycardia-induced myolysis and oxidative stress, thereby reducing the risk of AF [15]. However, the specific pathways involved and the extent of their contribution to the potential protective effect remain to be elucidated. In contrast, the retrospective cohort study by Basnet et al. (2017) found no significant association between metformin use and the incidence of postoperative AF in diabetic patients undergoing cardiac surgery [16]. This discrepancy may be attributed to the specific population studied, as surgical patients often have advanced cardiovascular disease and multiple comorbidities, potentially obscuring the effects of metformin on AF risk. In addition, the acute perioperative state and associated inflammatory responses may influence the development of postoperative AF, potentially masking any protective effects of metformin [21,23].

The case report by Boolani et al. (2011) presented an intriguing finding, suggesting a potential link between metformin-induced lactic acidosis and recurrent episodes of AF [14]. While this observation is based on a single case, it highlights the importance of considering potential adverse effects and drug interactions when using metformin, particularly in patients with impaired renal function or other risk factors for lactic acidosis. Interestingly, the retrospective cohort study by Iqbal et al. (2022) found no significant difference in the risk of developing AF between patients initiated on metformin monotherapy and those on other non-insulin monotherapies for T2DM [18]. This finding suggests that the potential protective effect of metformin against AF may not be unique compared to other antidiabetic agents [24]. However, it is important to note that the study did not compare metformin to lifestyle interventions or placebo, limiting the interpretation of its specific effects on AF risk.

Several limitations should be considered when interpreting the findings of this systematic review. First, the included studies were observational in nature, precluding the establishment of a causal relationship between metformin use and AF risk. Unmeasured confounders, such as lifestyle factors, adherence to medication, and severity of comorbidities, may have influenced the observed associations. In addition, most studies did not report detailed information on metformin dosage, duration of use, or concomitant medications, which could potentially modify the effects on AF risk. Furthermore, the included studies were conducted in specific geographical regions (USA and Taiwan), limiting the generalizability of the findings to other populations with different genetic and environmental factors.

Despite these limitations, this systematic review highlights the potential for metformin to reduce the risk of AF in patients with T2DM. However, the strength of this association and the underlying mechanisms remain to be fully elucidated. Future research should focus on conducting well-designed prospective studies or randomized controlled trials to establish a causal relationship and explore the dose-response effects of metformin on AF risk. In addition, mechanistic studies investigating the pleiotropic effects of metformin on cardiac electrophysiology and atrial remodeling could provide valuable insights into the potential protective mechanisms. It is also crucial to consider the potential adverse effects and drug interactions associated with metformin use, particularly in patients with impaired renal function or at risk for lactic acidosis. Careful patient selection, dosage adjustment, and monitoring are essential to maximize the potential benefits while minimizing the risks.

## Conclusions

This systematic review investigated the relationship between metformin use and the risk of AF in patients with T2DM. The results varied across the included studies. Some research suggested a potential protective effect of metformin, linking its use to a reduced risk of developing AF. Proposed mechanisms for this effect include metformin's improvements in endothelial function, reductions in oxidative stress, and anti-inflammatory properties. However, other studies found no significant association, indicating that metformin may not uniquely protect against AF compared to other antidiabetic agents. Notably, some studies showed no reduction in postoperative AF incidence in patients undergoing cardiac surgery, suggesting that the perioperative state might influence outcomes. In addition, case reports highlighted the need to consider potential adverse effects, such as metformin-induced lactic acidosis potentially triggering AF. Given these mixed findings, further research, especially well-designed prospective studies or randomized controlled trials, is essential to establish a clear causal relationship and elucidate the underlying mechanisms of metformin's impact on AF risk in T2DM patients.
